# Outer Membrane Protein U (OmpU) Mediates Adhesion of *Vibrio mimicus* to Host Cells via Two Novel N-Terminal Motifs

**DOI:** 10.1371/journal.pone.0119026

**Published:** 2015-03-05

**Authors:** Xueqin Liu, Huihui Gao, Nin Xiao, Yan Liu, Jinnian Li, Lin Li

**Affiliations:** Key Laboratory of Zoonoses, College of Animal Science and Technology, Anhui Agricultural University, Hefei, P. R. China; CNR, ITALY

## Abstract

*Vibrio mimicus* (*V.mimicus*) is a causative agent of ascites disease in aquatic animals. Our previous studies have demonstrated that the outer membrane protein U (OmpU) from *V.mimicus* is an immunoprotective antigen with six immunodominant linear B-cell epitopes. Although the N-terminus of OmpU contains potential binding motifs, it remained unclear whether OmpU possesses adhesion function. Here, the adhesive capacity of recombinant OmpU and *V.mimicus* to epithelioma papulosum cyprinid (EPC) cells was determined by immunofluorescence and adherence assay. The results showed that after co-incubated with rOmpU, an obvious visible green fluorescence could be observed on the EPC cell surface and the nuclei exhibited blue fluorescence; while the control cell surface did not show any signal, only nuclei exhibited blue fluorescence. The average number of wild-type strain adhered to each cell was 32.3 ± 4.5. The average adhesion number of OmpU gene deletion mutant was significantly reduced to 10.8 ± 0.5 (P < 0.01) and restored to 31.3 ± 2.8 by complement strain (P >0.05). Pretreatment of cells with rOmpU reduced the average adhesion number of wild-type strain to 9.7 ± 2.9 (P < 0.01). Likewise, binding was significantly decreased to 8.8 ± 3.2 (P < 0.01) due to blocking role of OmpU antibodies. To determine binding motifs of OmpU, six immunodominant B-cell epitope peptides labeled with FITC were employed in flow cytometry-based binding assay. Two FITC-labeled epitope peptides (aa90-101 and aa173-192) showed strong binding to EPC cells (the fluorescence positive cell rate was 99 ± 0.6% and 98 ± 0.3%, respectively), which could be specifically competed by excess corresponding unlabeled peptides, whereas the remaining four showed a low level of background binding. This is the first demonstration that OmpU possesses adhesion function and its N terminal 90–101 and 173–192 amino acid regions are critical sites for cell surface binding.

## Introduction

*Vibrio mimicus* (*V*.*mimicus*), which resembles *Vibrio cholera*, is a common pathogen for humans and aquatic animals [1]. It is not only a causative agent of severe ascites disease in aquatic animals, but is also responsible for human bacterial gastroenteritis and food poisoning as a result of consumption of infected aquatic animal products [2–4]. Anti-microbial therapy is the primary means for controlling ascites disease. However, due to inappropriate use and abuse of antibiotics in aquaculture industry, more and more drug resistant *V*.*mimicus* strains emerged, which led to the death of large numbers of aquatic animals. These serious impacts require new means of ascites disease prevention in aquaculture industry.

Bacterial adhesion is the specific binding of the bacteria to the receptor on the epithelial cell surface of the host through adhesin. The receptor triggers a range of signaling pathways that lead to bacterial invasion and colonization. This process is a key step in the bacterial ability to evade the host immune system thus successfully establishing bacterial infection [5]. Collectively, adhesin refers to a variety of bacterial cell-surface structures that facilitate adhesion. Typically, pathogens have the ability to express an array of different adhesins. The identification of major adhesins and their critical functional domains creates opportunities for developing new strategies for effectively combating bacterial diseases, for example by creating adhesion antagonists and adhesin vaccines that block the initial bacterial infection [6].

Outer membrane protein U (OmpU) is a conserved major outer membrane protein, which is widely present in pathogenic vibrio, such as *Vibrio cholerae*, *Vibrio alginolyticus* and *Vibrio vulnificus*. OmpU protein was shown to be an important adhesin in *Vibrio cholerae* and *Vibrio vulnificus* [7–10]. However, in 1992, Uchimura et al. first reported that pili is the adhesin for *V*.*mimicus* [11], while in 1997, Alam et al. reported that OmpHA is the adhesin in *V*. *mimicus* [12–13]. Subsequently, Singh et al. [14] and Sumio et al. [15], using hexaplex PCR detection, demonstrated that *V*.*mimicus* also carries *OmpU* gene, and inferred that the OmpU protein may be a non-pilus adhesin in *V*.*mimicus*. In our previous study, six immunodominant linear B-cell epitopes of the OmpU protein from *V*.*mimicus* were identified [16]. We also found that OmpU protein was highly homologous to the corresponding sequence of *V*.*cholerae*, and the N-terminus of the OmpU protein in *V*.*mimicus* contained motifs of the outer membrane protein adhesin of Haemophilus [17], and anti-OmpU antibodies could inhibit the adhesion of *V*. *mimicus*. But the adhesion function of OmpU protein is unknown. The purpose of this study was to employ epithelioma papulosum cyprinid (EPC) cells as a cell model to address whether the OmpU protein has adhesion function and to identify the sites critical for receptor binding as a prelude to the potential development of adhesion antagonists and adhesin vaccines against *V*.*mimicus*.

## Materials and Methods

### Ethics statements

The bacterial strains used in this study were isolated or constructed in our previous studies[18]. The EPC cell line was obtained from the Ministry of Agriculture Fisheries Pathogenic Library. Rabbit antibodies specific for OmpU protein and the preimmune serum were available from previous studies [19], and other materials were bought from legal biotechnology companies. This study protocol was approved by the Ethics Committee of Anhui Agricultural University.

### Bacterial strains and growth conditions

*V*. *mimicus* strain 04–14 was isolated from fish with ascites disease. Presumptive *V*. *mimicus* colonies were identified by the API 20 NE system (BioMerieux, France) in accordance with the manufacturer’s protocol, and then confirmed by 16 S rRNA gene sequencing. Briefly, 20 ng of bacterial genomic DNA was used as a template to amplify the 16S rRNA gene, and the following amplification profile: initial denaturation at 94°C for 5min, followed by 30 cycles of denaturation at 94°C for 30 s, annealing at 52°C for 50 s, and extension at 72°C for 100 s. The two DNA primers used corresponded to the following positions in the *V*.*mimicus* ATCC 33653T 16S- rRNA gene sequence (GenBank accession no.: X74713.1): sense primer positions 6–25 (5′-AGAGTTTGATCCTGGCTCAG-3′); antisense primer positions 1434–1453 (5′- CCGAAGGTTAAACTACCTGC-3′). The obtained PCR fragments were sequenced by GenScript Biotechnology (Nanjing, China). Sequence analysis showed that the 16S rRNA gene sequence of the strain 04–14 was 1448 bp in length and its similarity with *V*.*mimicus* ATCC33653T was 99.4%. *V*.*mimicus* 04–14ΔOmpU deletion mutant and complemented strain were constructed in our previous studies [18]. *V*. *mimicus* strains were cultured in brain heart infusion broth (BHI; Beijing Solarbio Science & Technology Co., Ltd., China) at 30°C, while E. coli BL21 (DE3) was cultured in the Luria Bertani medium (LB medium; Beijing Solarbio Science & Technology Co., Ltd., China) at 37°C.

### Epithelioma papulosum cyprini (EPC) cells culture

EPC cell line was selected as the representative cell to examine the adhesion capacity of OmpU protein and *V*. *mimicus*. EPC cells were maintained in M199 medium (Wisent Inc. Nanjing, China) with conventional antibiotics and 10% fetal bovine serum (Gibco, USA) at 28°C in an incubator with 5% CO_2_ atmosphere. EPC cells grown in 25 cm^2^ culture flasks (Corning, NY, USA) were trypsinized and splitted into fresh medium at a volume ratio of 1: 4 at least once a week.

### Expression and purification of recombinant OmpU protein

The gene encoding OmpU was amplified from *V*.*mimicus* genomic DNA by PCR using the following primers: 5′- CGGGATCCGGCATTAACCAAAGTGGTGAC-3′ (*Bam*HI site underlined) and 5′- CCCAAGCTTTTAGAAGTCGTAACGTAGACC-3′ (*Hind*III site underlined). Purified PCR product and plasmid pET-32a were digested with *Bam*HI and *Hind*III, and then a ligation reaction was set up. The positive recombinant plasmid was transformed into *E*. *coli* BL21 (DE3) competent cells. The recombinant *E*. *coli* BL21 (DE3) containing the plasmid pET-32-OmpU was inoculated into LB liquid medium with 100 μg/ml ampicillin, followed by incubation at 37°C overnight with constant shaking. Subsequently, the culture was inoculated into 200 mL of LB liquid medium at a volume ratio of 1:100 and incubated at 37°C for 3 h with shaking. When the bacterial culture entered the logarithmic growth phase, isopropyl-β-D-thiogalactopyranoside (IPTG; Sigma, USA) was added at a final concentration of 0.5 mmol/L to induce protein expression at 37°C for 3 h. The bacterial cells were harvested by centrifugation at 10,000 × g for 5 min at 4°C. After analysis by sodium dodecyl sulfate-polyacrylamide gel electrophoresis (SDS-PAGE), the soluble recombinant OmpU (rOmpU) was purified on Ni-NTA resin (GE Healthcare, Piscataway, NJ, USA) according to the manufacturer’s instructions, and the concentration of purified protein was determined by a Bradford Protein Assay Kit (Tiangen, Beijing, China). In addition, Western blotting was performed with purified OmpU-specific antibody produced in our previous studies[19] to confirm the immunoreactivity of the rOmpU.

### Detection of adhesive capbility of rOmpU protein by immunofluorescence assay

EPC cells were cultivated in 24-well tissue culture plates (Corning, NY, USA) containing sterile glass coverslips (Corning, NY, USA) to 80% confluence.Cells then were washed 3 times with phosphate-buffered saline (PBS), before being fixed with 4% paraformaldehyde for 15 min at room temperature. After three washes with PBS, cells were permeabilized with 1% (v/v) Triton X-100 (500 μL / well) for 15 min. Cells were washed 3 times in PBS, blocked for 1 h with 3% bovine serum albumin (BSA; Sigma), and rinsed 3 times in PBS again. 200μL of the purified rOmpU protein at a concentration of 500 μg/mL was added into each well (repeat three wells). Following incubation for 1 h at 28°C and 3 rinses in PBS, 200μL purified rabbit anti-OmpU antibody was added at a dilution of 1:200. Samples were incubated for 1 h at 28°C, followed by 3 washes in PBS. 100μL of a 1:100 dilution of FITC (fluorescein isothiocyanate)-labeled goat anti-rabbit IgG (Jackson ImmunoResearch Laboratories, Inc. USA) was added into each well and incubated in the dark for 1 h. After 3 washes in PBS, 50 μL DAPI (4, 6-Diamidino-2-phenylindole dihydrochloride; Ziyi reagent Co., Shanghai, China) staining solution was added into each well and nuclei was stained in the dark for 10 min at 28°C. After thorough washing, the coverslips were taken out and the binding of the OmpU protein to EPC cells was observed under a fluorescence microscope. EPC slides with M199 culture fluid instead of rOmpU were processed likewise and used as controls to exclude that cell surface staining is due to non-specific reactivity of the primary and second antibody. Experiment was done in triplicate.

### Adherence and adherence-inhibition assay

To measure the adhesive capacity of *V*.*mimicus* strains (wild-type strain, Δ*OmpU* deletion mutant and its complemented strain) to EPC cells, cells were seeded onto glass coverslips in 24-well plates at a density of 10^5^ cells per well, and grown for 24 h at 28°C with 5% CO_2_. The medium was replaced with RPMI-1640 medium containing 2% FCS before infection. *V*.*mimicus* suspension with an MOI (MOI, number of bacteria per cell) of 100:1 was added to the EPC monolayers in 1 mL culture medium. Bacteria were allowed to adhere for 60 min at 28°C in 5% CO_2_. After incubation, each well was rinsed five times with PBS and fixed with pre-cooled methanol for 10 min, stained with Crystal Violet, and examined under a light transmission microscope at a magnification of ×1,000 (oil immersion). Fifty cells were randomly selected to count the number of adhering bacteria per slide. Slide with EPC cells, but no bacteria was served as control. Adhesion assay were repeated three times, each sample repeated three cell wells.

To further examine the effect of OmpU protein and its antibody on *V*. *mimicus* adhesion, M199 basic medium without serum and antibiotics was used to dilute rOmpU protein to a final concentration of 50 μg/mL. 500 μL of the protein solution was added to 24-well cell culture plates with glass coverslips, whereas negative control received culture medium alone. Cell culture plates were incubated at 28°C for 1 h, rinsed three times with PBS, and 500μL of *V*. *mimicus* strain 04–14 suspension (2 × 10^7^ CFU / mL counted on BHI agar plate) was added to each well, the rest of the procedure was the same as above. Similarly, anti-OmpU antibody and the preimmune serum (negative control) at a dilution of 1:200 dilution was added to a suspension of *V*. *mimicus* strain 04–14 (2 × 10^7^ CFU / mL) and incubated for 1h at 28°C. The mixture was tested for bacterial adhesion exactly as described above. Each experiment was performed in triplicates, each sample repeated three cell wells.

### Identification of OmpU motifs critical for cell surface binding

In order to determine the OmpU protein motifs critical for cell surface binding, six immunodominant B cell epitope peptides of the OmpU protein (Table 1) and an unrelated scrambled-sequence peptide were synthesized by Yao Qiang Shanghai Biological Technology Co. These peptides were labeled with FITC at their N-terminal and purified by high performance liquid chromatography to a purity of 98%. EPC cell monolayers grown to about 80% confluency in culture flasks were trypsinized and diluted to a concentration of 1 × 10^6^ cells/mL. 1 mL each was dispensed into 9 ethylene propionate tubes. 6 of the tubes received 100 μl of each of the six FITC-labeled epitope peptides at a concentration of 25 μg/mL. The remaining tubes received equal volumes of FITC-labeled scrambled-sequence peptide (25 μg/mL), unconjugated FITC dye (fluorescein control) and PBS (blank control), respectively. The sample tubes were left in dark for 60 min at 28°C. Cells were washed three times in PBS, re-suspended and fixed in 500 μL 1% paraformaldehyde solution, filtered through a 300 micron nylon mesh, and analyzed by flow cytometry to determine whether the epitope peptides bound specifically to EPC cells in vitro. Meanwhile, competition binding between FITC-epitope peptide and respective epitope peptide to EPC cells was performed. Briefly, 1mL of EPC cells suspended at 1 × 10^6^ cells / mL were mixed with 100 μL of FITC-labeled epitope peptides (25μg/mL) and an equal volume of the unlabeled peptides at various concentrations (0, 50, 500, 1000 μg/mL). Cells were incubated for 60 min at 28°C in the dark, the rest of the procedure was the same as above. Each experiment was performed in triplicates.

**Table 1:** Amino acid sequenc of linear B-cell epitope peptide of OmpU protein.

*Epitope peptide no*.	*Amino acid position* ^*a*^	*Peptide sequence*	*Average antigenic index*
Epitope peptide_25–36_	25 to 36	NQSGDKAGSTVY	2.33
Epitope peptide_90–101_	90 to 101	FTTADNDSGSDL	2.58
Epitope peptide_115–126_	115 to 126	GEVTYGKNDGSL	2.51
Epitope peptide_173–192_	173 to 192	RFADRDTSTGEFADNKEDGY	2.83
Epitope peptide_211–222_	211 to 222	YADQNDNNEYML	2.02
Epitope eptide_239–250_	239 to 250	DGEKNFDSNSNG	2.49

^a^The corresponding position of each peptide in the native OmpU protein.

### Statistical analysis

The data obtained from adherence and adherence-inhibition assay were expressed as the mean number of adhensive bacteria per cell ±SEM., and subjected to one-way of variance (ANOVA) followed by Duncan’s test to determine differences in adhesive capacity among different strains and different treatment groups. Statistical significance of the fluorescence positive cell rate between epitope peptide groups and control groups was demonstrated using the student’s t test. Differences were considered to be statistically significant at P<0.05. Statistical analyses were performed using SAS for Windows version 9.0 (SAS, Chicago, USA).

## Results

### Purification and confirmation of recombinant OmpU protein

**Fig 1:**
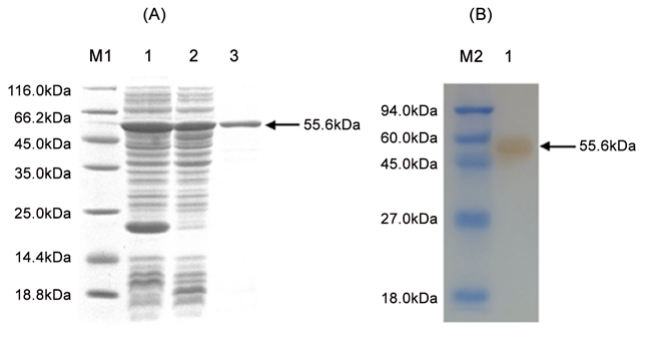
SDS-PAGE and Western blot analysis of rOmpU expressed in *E*. *coli* BL21 (DE3). (**A**) M1, protein molecular weight standard; Lane 1, whole cell bacterial lysate after induction of pET-32a-OmpU/BL21 (DE3); Lane 2, soluble fraction part of the baterial lysate; Lane 3, purified rOmpU. (**B**) M2, protein molecular weight standard; Lane 1, Western blot of the purified rOmpU with OmpU-specific antibodies.

To obtain rOmpU, the recombinant *E*. *coli* BL21 (DE3) cells harboring the plasmid pET-32-OmpU were induced with IPTG. SDS-PAGE analysis revealed a 55.6 kDa soluble protein band appeared in the original and purified expression products (Fig. 1A), and its size was consistent with the theoretically expected relative molecular weight of the His-OmpU protein. The yield of rOmpU was ~10mg/L bacterial culture. Western blot analysis of the purified protein with OmpU-specific antibodies revealed a single band at about 55.6 kDa (Fig. 1B). These results confirm the identity of the recombinantly expressed protein as represented by OmpU.

### The adhesion function of OmpU protein

**Fig 2:**
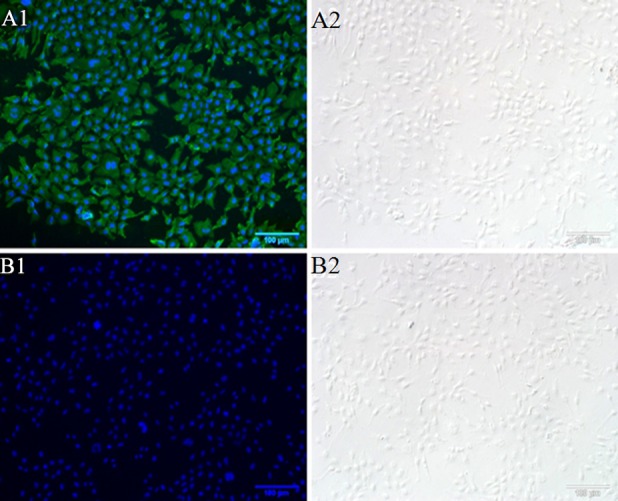
Binding of the OmpU protein to EPC cells determined by immunofluorescence. (**A**) EPC cells were co-incubated with the rOmpU protein, followed by washing and visualization of surface retained rOmpU by immunofluorescent staining with OmpU-specific antibodies (green color). Nuclei was counterstained with DAPI (blue color). Bright field images were shown for reference. (**B**) EPC cells were co-incubated with M199 medium instead of rOmpU. Same staining as in (A). All scale bars are 100μm.

In order to investigate whether OmpU protein has adhesion function, we developed an fluorescence-based adhesion assay. Upon incubation of EPC cells with rOmpU, cell surface attachment of OmpU protein was assessed with anti-OmpU primary antibody followed by FITC-labeled secondary antibody. DAPI was used to counterstain cell nuclei. High green fluorescence staining could be detected on the surface of EPC cells co-incubated with rOmpU (Fig. 2A), whereas control cells, which were incubated with M199 medium instead of rOmpU and were stained with primary antibody and FITC-labeled secondary antibody, did not emit any green signal (Fig. 2B).

**Fig 3:**
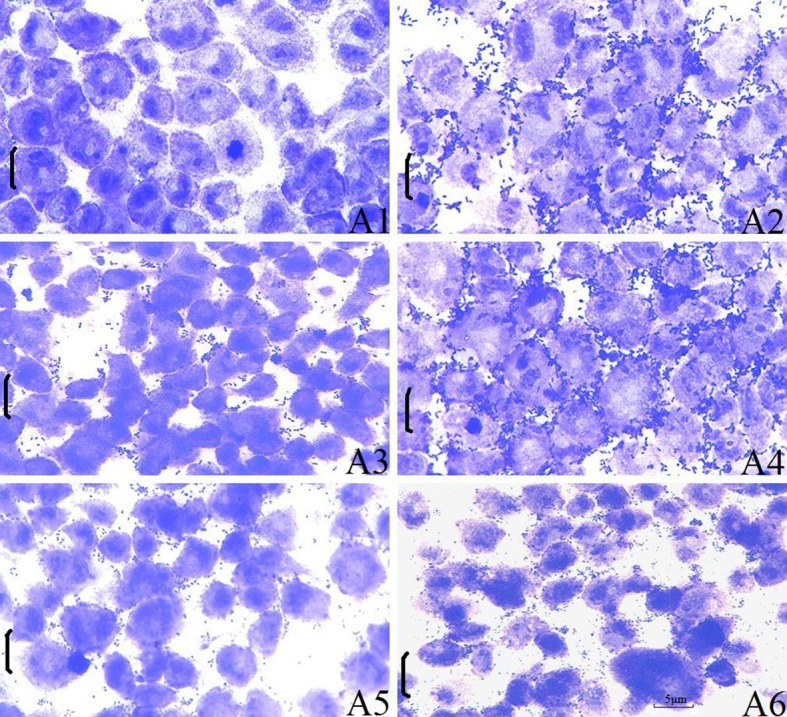
Adhesion of *V*.*mimicus* to EPC cells. Micrographs of EPC cells exposed to various bacterial strains and stained with Crystal Violet. (**A1**) control EPC cells, (**A2**) adhesion of wild-type strain to EPC cells, (**A3**) adhesion of Δ*OmpU* deletion mutant to EPC cells, (**A4**) adhesion of complemented strain to EPC cells, (**A5**) adhesion of wild-type strain after pre-treating EPC cells with rOmpU, (**A6**) adhesion of wild-type strain to EPC cells after pre-treating bacteria with OmpU-specific antibodies. Magnification 1,000 x. All scale bars are 5μm.

To determine the role of OmpU in the adherence of *V*.*mimicu*s to EPC cells, the adhesive level of wild-type strain, Δ*OmpU* deletion mutant and complemented strain, which have similar growth rate under the conditions employed in this assay, was examined by counting the number of viable bacterial cells adhered to the EPC cells monolayer at the end of the incubation period. As shown in Fig. 3A2, wild-type strain exhibited an aggregative adherence to EPC cells, whereas control cells showed neither bacterial adhesion nor cytopathic effects (Fig. 3A1). The average number of wild-type strain, Δ*OmpU* deletion mutant and complemented strain adhered to each cell was 32.3 ± 4.5, 10.8 ± 0.5 and 31.3 ± 2.8, respectively. There was a significant decrease in the level of adhesion of Δ*OmpU* deletion mutant (Fig. 3A3) compared to wild-type strain (P<0.01), which could be restored by complemented strain (Fig. 3A4). To directly address the role of OmpU in the binding process, adherence-inhibition assay using anti-rOmpU antibodies and rOmpU protein was performed. Both pre-incubation of EPC cells with rOmpU and pre-treatement of wild-type strain with anti-rOmpU antibodies significantly blocked the bacterial adhesion (Fig. 3A5–6). The former reduced the average adhesion number of wild-type strain to 9.7 ± 2.9 (P < 0.01), whereas the latter decreased to 8.8 ± 3.2 (P < 0.01). These results reveal both the importance and sufficiency of OmpU in mediating the adherence of *V*.*mimicu*s to EPC cells.

### Identification of OmpU motifs critical for adherence

**Fig 4:**
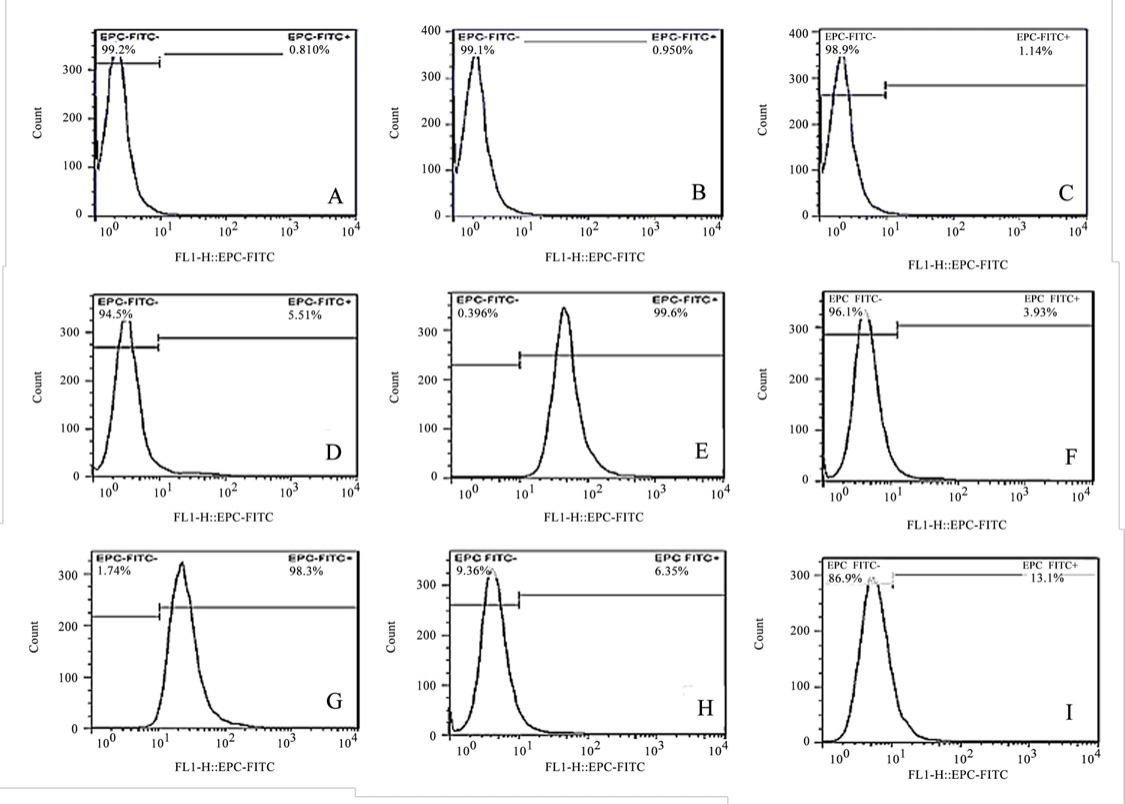
Binding of OmpU derived epitope to EPC cells as determined by flow cytometry. EPC cells were co-incubated with (**A**) PBS, (**B**) an irrelevant FITC-labeled peptide, (**C**) unconjugated FITC dye, FITC-labeled epitope peptide 25–36 (**D**), 90–101(**E**), 115–126 (**F**), 173–192 (**G**), 211–222 (**H**), 239–250 (**I**), and cell surface binding was assessed by flow cytometry.

**Fig 5:**
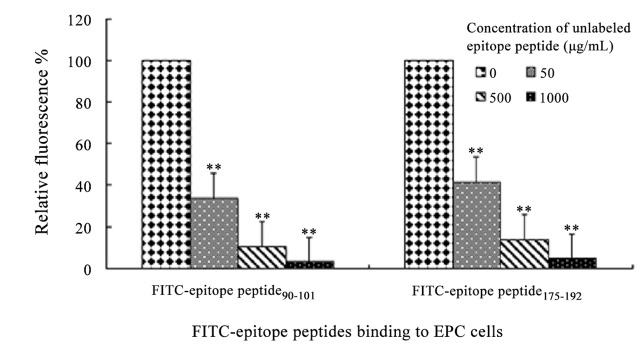
Competition of labled epitope peptide-EPC cells interactions by unlabeled peptide. Fluorescence positive cell rate after the EPC cells co-incubated with labled epitope peptide was initialized to 100%. Labled epitope peptide 90–101 reduced by 58.6%, 86.3% and 95.4% binding to EPC cells, respectively, with the concentration increasing of corresponding unlabeled peptide from 50 to 1000 μg/mL, whereas labeled epitope peptide 173–192 reduced by 58.6%, 86.3% and 95.4% binding. Data are representative of three experiments.

To determine the key binding motifs of OmpU protein, flow cytometry-based binding assay was used to examine the binding of FITC-labeled epitope peptide to EPC cells. As shown in Fig. 4, the fluorescence positive cell rate was 5±0.5%, 99±0.6%, 3±0.9%, 98±0.3%, 6±0.4% and 13±0.1% in the EPC cells co-incubated with six labeled epitope peptides, respectively. Negligible cell labeling was observed with the negative controls, which included PBS (0.8±0.04%), labeled scrambled-sequence peptide (0.9±0.05%), and unconjugated FITC dye (1± 0.1%). Two of these epitope peptides (Fig. 4E, G), namely, epitope peptides 90–101 and 173–192, led to significant fluorescence cell labeling over negative controls (P <0.01). To assess the specificity of cell surface binding of the two epitope peptides, an excess of the corresponding unlabeled peptide was used in competition experiment. Flow cytometry showed that excess unlabeled epitope peptides competitively inhibited labeled peptides binding with cell surface in a concentration dependent manner. Compared to the control level (no added unlabeled epitope peptide), excess unlabeled epitope peptide 90–101 (50, 500 and 1000 μg/mL) respectively led to a 66.4%, 89.8% and 97.1% reduction in the fluorescence positive cell rate (P<0.01), whereas excess unlabeled epitope peptide 173–192 (50, 500 and 1000 μg/mL) led to a 58.6%, 86.3% and 95.4% reduction (P<0.01), respectively (Fig. 5). These results suggested that epitope peptides 90–101 and 173–192 are critical binding motifs of OmpU protein that mediate specific cell surface binding.

## Discussion

To our knowledge, this study is the first report to confirm that the *V*.*mimicus* OmpU protein possesses adhesion function through cell adhesion model. Our study therefore provides a foundation for further research on adhesion antagonists and adhesin vaccine directed against *V*. *mimicus*, as well as the role of the OmpU protein in pathogenesis of *V*.*mimicus*.

EPC cell line, derived from carp papillary epithelial tumor cells, are rapidly proliferating adherent cells [20]. The epithelial cell line is routinely used to evaluate adherence potential of aquatic animal pathogens in vitro, including *Edwardsiella tarda*, *Vibrio anguillarum*, *Acinetobacter spp*, and *Aeromonas hydrophila*, because it is likely to be the first cell type that a fish pathogen would encounter in vivo before establishing infection [21–26]. *V*. *mimicus*, like *V*.*cholerae*, is gastrointestinal pathogen. Strain 04–14 was isolated from liver and intestine of carp with ascites disease. However, it is difficult to assess bacteria or protein adhesion to intestinal cells in vivo. Likewise, due to instability, primary cultures of carp intestinal epithelial cells present a poor option. Hence, we still selected EPC cell line as the adhesion model to determine the adhesive capacity of the *V*.*mimicus* strain 04–14 and OmpU protein in vitro. Despite the advantages of the EPC cells for bacterial adhesion studies, we found poor adherence of these cells to culture flasks and glass coverslips in our research, a limitation that was circumvented by precoating culture dishes with poly-lysine. Precoating not only improved EPC adhesion, it also reduced non-specific binding of protein reagents added to the cultures. Through thorough optimization, we arrived at conditions that allowed reliable measurement of bacterial adhesion to EPC cells, i.e. the optimal bacteria/cell ratio was maintained at 100:1, whereas appropriate incubation temperature and time were 28°C and 60 min, respectively. Under these conditions, EPC cells showed no change in morphology, and *V*.*mimicus* exhibited a distinct aggregative adherence to EPC cells. The successful establishment of the *V*. *mimicus* adhesion model laid the foundation for the subsequent functional studies.

It has previously been demonstrated that OmpU is an important adhesin in *V*.*cholerae* [9]. Considering that *V*. *mimicus* is closely related to *V*. *cholerae* due to sharing virulence factors, such as enterotoxins and hemolysins [14,27], in the present study we hypothesized that OmpU protein may also be the adhesin of *V*. *mimicus* and provided evidence that the *V*.*mimicus* OmpU protein indeed has adhesion function. This conclusion is supported by the following findings: first, rOmpU protein of *V*. *mimicus* could specifically bind to the EPC cell surface. Second, both rOmpU protein and OmpU-specific antibody could restrain the *V*. *mimicus* adherence to EPC cells. Finally, the adhesion of the *OmpU* gene deletion mutant to EPC cells was significantly decreased by 66.6% compared to wild-type strain, while the adhesion level of the complementary strain was restored, which thus suggested that the possibility of adhesion inhibition being caused by the antigen-antibody steric effect was excluded. Notably, both rOmpU protein and its antibody could not completely blocked strain 04–14 adherence to EPC cells in our study, suggesting a role of additional adhesins, such as pili adhesin and OmpHA adhesion reported by other scholars [11–12].

The binding motifs of the adhesin that mediate cell surface attachment are presumed critical functional regions of the protein, such as the epitope domain of protein. In our previous study, six immunodominant linear B-cell epitopes of OmpU protein were identified through epitope prediction followed by concrete experimental validation [16]. In the present study, we further identified whether these epitope peptides are critical binding domain of OmpU adhesin. It was found that two of six labeled epitope peptides, spanning residues 90–101 and 173–192 in the N terminal region of the protein, could bind to EPC cells surface examined by flow cytometry-based binding assay. We also found the excess corresponding unlabeled epitope peptides were capable of competitive inhibition of binding of the two labeled epitope peptides to EPC cells in a concentration dependent manner. This phenomenon is consistent with the characteristics of receptor-ligand binding competitive inhibition[28–29], indicating the binding the two epitope peptide to EPC cells was specific. Taken together, these results reveal that epitope peptides 90–101 and 173–192 are two novel functional motif associated with adherence.

## Conclusions

This is the first report to demonstrate that the OmpU protein derived from *V*. *mimicus* possesses adhesion function and to uncovered two novel N-terminal motifs of OmpU mediating specific cell surface attachment of *V*. *mimicus*. This study may contribute to design and creat new adhesion antagonists and adhesin vaccine against *V*. *mimicus*.
